# [^68^Ga]Ga‑PSMA‑617 PET-based radiomics model to identify candidates for active surveillance amongst patients with GGG 1–2 prostate cancer at biopsy

**DOI:** 10.1186/s40644-024-00735-2

**Published:** 2024-07-04

**Authors:** Jinhui Yang, Ling Xiao, Ming Zhou, Yujia Li, Yi Cai, Yu Gan, Yongxiang Tang, Shuo Hu

**Affiliations:** 1 Department of Nuclear Medicine, Xiangya Hospital, Central South University, 87 Xiangya Road, Changsha, Hunan 410008 China; 2 Department of Urology, Disorders of Prostate Cancer Multidisciplinary Team, Xiangya Hospital, Central South University, 87 Xiangya Road, Changsha, Hunan 410008 China; 3 National Clinical Research Center for Geriatric Disorders (XIANGYA), Xiangya Hospital, Central South University, Changsha, Hunan China; 4 Department of Nuclear Medicine, Inselspital, University Hospital Bern, Bern, Switzerland; 5 Key Laboratory of Biological, Nanotechnology of National Health Commission, Xiangya Hospital, Central South University, Changsha, Hunan China

**Keywords:** Prostate cancer, Active surveillance, Positron-emission tomography, Radiomics

## Abstract

**Purpose:**

To develop a radiomics-based model using [^68^Ga]Ga-PSMA PET/CT to predict postoperative adverse pathology (AP) in patients with biopsy Gleason Grade Group (GGG) 1–2 prostate cancer (PCa), assisting in the selection of patients for active surveillance (AS).

**Methods:**

A total of 75 men with biopsy GGG 1–2 PCa who underwent radical prostatectomy (RP) were enrolled. The patients were randomly divided into a training group (70%) and a testing group (30%). Radiomics features of entire prostate were extracted from the [^68^Ga]Ga-PSMA PET scans and selected using the minimum redundancy maximum relevance algorithm and the least absolute shrinkage and selection operator regression model. Logistic regression analyses were conducted to construct the prediction models. Receiver operating characteristic (ROC) curve, decision curve analysis (DCA), and calibration curve were employed to evaluate the diagnostic value, clinical utility, and predictive accuracy of the models, respectively.

**Results:**

Among the 75 patients, 30 had AP confirmed by RP. The clinical model showed an area under the curve (AUC) of 0.821 (0.695–0.947) in the training set and 0.795 (0.603–0.987) in the testing set. The radiomics model achieved AUC values of 0.830 (0.720–0.941) in the training set and 0.829 (0.624–1.000) in the testing set. The combined model, which incorporated the Radiomics score (Radscore) and free prostate-specific antigen (FPSA)/total prostate-specific antigen (TPSA), demonstrated higher diagnostic efficacy than both the clinical and radiomics models, with AUC values of 0.875 (0.780–0.970) in the training set and 0.872 (0.678–1.000) in the testing set. DCA showed that the net benefits of the combined model and radiomics model exceeded those of the clinical model.

**Conclusion:**

The combined model shows potential in stratifying men with biopsy GGG 1–2 PCa based on the presence of AP at final pathology and outperforms models based solely on clinical or radiomics features. It may be expected to aid urologists in better selecting suitable patients for AS.

**Supplementary Information:**

The online version contains supplementary material available at 10.1186/s40644-024-00735-2.

## Introduction

Active surveillance (AS) is the recommended strategy for patients with low-risk, localized prostate cancer (PCa) and is being recommended for some intermediate-risk patients [1, 2]. It aims to postpone or avoid active treatment in individuals with localized PCa, while maintaining their quality of life and functional outcomes, and reducing overtreatment [1, 3]. However, the lack of consensus on inclusion criteria stringency and disease progression definition has led to significant variability in AS protocols across different centers and guidelines [4, 5]. Consequently, the cumulative five-year dropout rate on AS reaches 44%, with 27% triggered by disease progression [6, 7]. Given the potential for tumor progression and metastasis during AS, determining optimal selection criteria remains a crucial issue.

According to established clinical criteria and indicators, including the prostate-specific antigen (PSA), clinical T-stage, and biopsy findings, current guidelines classify patients with localized PCa into risk categories and recommend AS for all low-risk patients [8]. Furthermore, AS has been proposed as an option for selected intermediate-risk PCa patients with low-volume Gleason Grade Group (GGG) 2. Several studies have demonstrated the oncologic safety of AS relative to aggressive treatment [9–11]. However, biopsy results tend to underestimate the actual GGG of patients, resulting in some patients not meeting the enrollment criteria for receiving an AS regimen. Up to 25% of patients with biopsy GGG 1–2 PCa may qualify for AS but harbour adverse pathology (AP: pT3 and/or N1 and/or GGG ≥ 3) at radical prostatectomy (RP) [12, 13]. By enrolling these patients in AS, they may miss the opportunity for curative treatment due to disease progression [14, 15]. Additionally, the inflexibility of risk categories may limit the number of patients with pathologically indolent PCa who qualify for AS, increasing the risk of overtreatment [16]. Recently, several multivariate models and nomograms based on clinical and multiparametric magnetic resonance imaging (mpMRI) features have been developed to overcome these limitations and have shown superior diagnostic efficacy compared to traditional risk categories [17, 18]. However, the usefulness of these models in terms of diagnostic accuracy is still controversial, and prostate biopsies for confirming PCa still tend to rely on PSA-based specificity [19].

Positron emission tomography/computed tomography (PET/CT) with [^68^Ga]-labeled prostate-specific membrane antigen inhibitors ([^68^Ga]Ga-PSMA) has been widely used in the clinical staging of primary PCa and the restaging of biochemically recurrent PCa [20, 21]. Previous studies have demonstrated a strong positive correlation between the maximal standardized uptake value (SUVmax) of [^68^Ga]Ga-PSMA PET and GGG for primary prostate tumors, indicating its potential to predict pathological upgrade from biopsy to RP [22]. The implementation of this novel molecular imaging technique could prove more advantageous than mpMRI in the patient screening process for AS [15]. In recent years, the field of radiomics has rapidly progressed, offering the ability to extract valuable quantitative data from digitally encrypted medical images, thereby providing additional information on lesions [23]. The combination of radiomics and machine learning has exhibited the capacity to accurately predict postoperative GGG of PCa in a non-invasive manner [24]. Notably, unlike tumor biopsies, radiomics has the potential to characterize the local tumor phenotype based on the entire lesion, rather than relying on tumor subsamples.

Our study aims to develop and validate a stratified machine learning model that combines [^68^Ga]Ga-PSMA PET/CT with traditional clinical risk factors. This model will be used to predict postoperative AP in patients with GGG 1–2 at biopsy, aiding in selecting patients for AS.

## Materials and methods

### Patients

The study protocol was approved by the Ethics Committee of Xiangya Hospital Central South University, and written informed consent to use the data was obtained from all included patients. We reviewed consecutive patients with biopsy GGG 1–2 PCa from April 2020 to May 2023. All enrolled men underwent transrectal ultrasound (TRUS)-guided biopsy and were treated with RP ± lymph node dissection in the Department of Urology, Xiangya Hospital. The exclusion criteria were as follows (a) patients who had prior PCa treatment before RP; (b) patients who had missing clinical data or nonstandard examinations; (c) patients who underwent a prostate biopsy with less than 12 cores taken. Overall, 75 patients were enrolled, and clinical features such as age, total PSA (TPSA), and free PSA (FPSA) were gathered for all selected patients.

### [^68^Ga]Ga-PSMA-617 PET/CT examination and image evaluation

[^68^Ga]Ga-PSMA-617 was administered to patients intravenously, with an activity of 3.7 − 4.44 MBq/kg. PET imaging was then performed after low-dose CT scanning at 40 ± 10 min post-injection. All scans were acquired using a PET/CT scanner (690 Elite; General Electric Healthcare). First, a CT scan (140 kV; 340 mAs; pitch, 1:1; slice thickness, 3.75 mm; matrix, 512 × 512) was performed from the head to mid-thigh for anatomical localization and attenuation correction. Next, PET scanning was performed, with 1.75 min per bed position. Finally, all PET images were reconstructed as a 256 × 256 transaxial matrix using the 3-dimensional ordered-subsets expectation maximization algorithm with 2 iterations and 23 subsets. The voxel size of the PET images was 2.7 × 2.7 × 3.8 mm.

Two experienced nuclear medicine doctors who were blind to the pathological outcomes evaluated the [^68^Ga]Ga-PSMA-617 PET/CT images independently, with disagreements being settled through discussion. Any focal uptake above the background in the prostate that couldn’t be explained by physiological uptake was considered a positive lesion. The SUVmax, the mean SUV (SUVmean), and PSMA tumor volume (PSMA-TV) were produced automatically from the volume of interest (VOI) with isocontours set at 41% of the maximum uptake within the respective focus [25, 26]. PSMA total lesion (PSMA-TL) was calculated by multiplying the PSMA-TV and SUVmean.

### Histopathology examination

All patients participating in the study underwent TRUS-guided biopsies under the supervision of an experienced urologist (Y Cai), who had performed over 1000 TRUS biopsies. RP ± pelvic lymph node dissection was subsequently performed for all patients with biopsy-proven PCa in this study using a laparoscopic approach. All of the slides from prostate biopsies and RP specimens were examined by a single, experienced uropathologist. Biopsy and pathological GGG were determined according to the 2019 International Society of Urologic Pathology (ISUP) criteria [27]. Prostatectomy pathological T stage (pT) and N stage (pN) were evaluated according to American Joint Committee on Cancer (AJCC) guidelines [28]. The presence of adverse pathology at RP, which is defined as non-organ confined disease (pT3) and/or lymph node invasion (pN 1) and/or GGG ≥ 3, represented the study's outcome, which means that the patient wasn’t suitable for AS. For patients who were under consideration for inclusion in AS protocols, this represents a surrogate endpoint for stronger oncological outcomes.

### Image segmentation

The whole prostate was manually delineated using 3D Slicer (vision 5.3.0) by two experienced nuclear medicine physicians. The entire prostate was utilized as the VOI because it avoids sampling error, radiomics problems for small lesions, and the challenges of multi-lesion characterization. It also offers a more robust inter-reader reproducibility and better accounts for tumor heterogeneity. Considering the low anatomical accuracy of PET imaging, we drew the VOI of the whole prostate on the CT images and then matched it to the PET images. To ensure accurate matching of PET and CT, manual transformation of PET images in 3 axes was allowed and the panes of PET images were interpolated to match the CT in our study. If necessary, VOI would be adjusted to prevent the inclusion of bladder activity.

### Radiomics features extraction and selection

The Radiomics features were extracted from the VOIs using the Pyradiomics platform (version 3.7.4) implemented in Python (version 3.7.4), according to the guidelines of the Image Biomarker Standardization Initiative [29]. All VOIs were normalized, discretized using fixed bin width (FBW = 0.25), and then resampled to 2.0 × 2.0 × 2.0 mm^3^ voxels before feature extraction. A total of 107 3D radiological features were extracted, which were categorized into seven feature classes: shape (*n* = 14), first order (*n* = 18), Gray Level Co-occurrence Matrix (GLCM) (*n* = 24), Gray Level Dependence Matrix (GLDM) (*n* = 14), Gray Level Run Length Matrix (GLRLM) (*n* = 16), Gray Level Size Zone Matrix (GLSZM) (*n* = 16), and Neighbouring Gray Tone Difference Matrix (NGTDM) (*n* = 5).

After radiomics feature extraction, we used two steps to select the features. At first, the minimum redundancy maximum relevance (mRMR) algorithm, which has been proven to be effective in radiomics feature selection [30], was performed to eliminate the redundant and irrelevant features. Then, the least absolute shrinkage and selection operator (LASSO) regression model, was conducted to choose the optimized subset of features to construct the final model.

### Model construction

For model development and assessment, patients were randomly divided into a training and testing group in a ratio of 7:3. All models were developed based on the training cohorts and subsequently evaluated on the testing cohorts.

The clinical model was constructed in two steps based on the clinical features. Firstly, univariate logistic regression was performed to assess clinical features including age, PSA, free PSA (FPSA), prostate volume, PSA density (PSAD), FPSA/total PSA (TPSA), biopsy GGG, % of positive cores, SUVmax, SUVmean, PSMA-TL and PSMA-TV. Then, those features with *P* < 0.05 in univariate logistics analysis were analyzed in multivariate logistic regression analysis to build a model.

We used a logistic regression classifier to build the radiomics model based on the selected radiomics features. A stratified tenfold cross-validation was applied with 100 iterations in the training group to develop a reliable and stable model, and the model was then assessed in the testing group. Radiomics score (Radscore) was calculated for each patient via a linear combination of selected features that were weighted by their respective coefficients.

For the combined model, clinical features with *P* < 0.05 in univariate logistics and Radscore were imported into the multivariate logistic regression, and statistically significant indicators were screened to establish a visualized quantitative model, the nomogram outcome stratification prediction model.

### Statistical analysis

The *t*-tests/Spearman rank tests and Chi-square/Fisher's exact tests were used to compare the clinical features between men with AP and those without. Univariate and multivariate logistic regression analyses were performed to determine independent predictors, and then build the prediction models.

The areas under the receiver operating characteristic (ROC), area under the curve (AUC), decision curve analysis (DCA), and calibration curve were used to assess the diagnostic value, clinical utility, and predictive accuracy of those models, respectively. Statistical analysis was performed using IBM SPSS statistics software, version 26.0, and R software, version 4.1.3. *P* < 0.05 was considered statistically significant.

## Results

### Patient characteristics

A total of 75 patients with biopsy GGG 1–2 PCa were included in this study. At final pathology after RP for all patients, 30 patients (40%) had AP. Thirty patients with AP at RP were randomly divided into the training cohort (*n* = 21) and the testing cohort (*n* = 9). Of the 45 patients without AP at RP, 32 patients were assigned to the training cohort and 13 patients were assigned to the test cohort. There were no significant differences in all clinical and image features between the training cohort and testing cohort. Table 1 shows the characteristics of all patients in detail.

**Table 1:** Patient characteristics

	Patients without AP at final pathology (*n* = 45)	Patients with AP at final pathology (*n* = 30)	*P* values	Training Cohort (*n* = 53)	Testing Cohort (*n* = 22)	*P* values
Mean age (SD), y	66.42 (7.88)	64.50 (6.7)	0.688	66.15 (7.38)	64.46 (7.62)	0.898
Median PSA (IQR), ng/ml	9.20 (5.80–13.92)	15.01 (11.14–25.50)	0.002*	12.89 (7.17–17.33)	9.52 (6.19–17.24)	0.120
Median FPSA (IQR), ng/ml	1.21 (0.58–1.91)	1.42 (0.81–2.27)	0.236	1.42 (0.76–2.18)	0.96 (0.55–1.60)	0.296
Median PV (IQR), ml	32.40 (21.45–32.40)	31.20 (22.15–52.75)	0.991	31.40 (20.20–47.60)	31.50 (25.53–42.20)	0.858
Median PSAD (IQR), ng/ml^2^	0.29 (0.17–0.45)	0.45 (0.24–0.67)	0.023*	0.38 (0.17–0.59)	0.34 (0.17–0.47)	0.858
Median FPSA/TPSA (IQR)	0.14 (0.10–0.18)	0.09 (0.07–0.14)	0.017*	0.13 (0.09–0.16)	0.10 (0.06–0.14)	0.233
Biopsy GGG, n (%)			0.220			0.465
1	26	13		29	10	
2	19	17		24	12	
% of positive cores, median (IQR)	0.17 (0.09–0.25)	0.25 (0.12–0.39)	0.206	0.17 (0.11–0.32)	0.17 (0.08–0.27)	0.809
SUVmax	6.20 (5.15–11.25)	9.90 (6.80–17.43)	0.003*	7.80 (5.85–12.55)	8.45 (4.90–13.65)	0.633
SUVmean	3.90 (3.20–6.45)	5.65 (4.08–9.73)	0.005*	4.40 (3.70–6.90)	4.55 (2.90–7.63)	0.976
PSMA-TL	13.31(6.39–21.49)	25.88 (16.00–41.09)	< 0.001*	10.52 (5.47–23.54)	16.60 (0.00–41.97)	0.743
PSMA-TV	1.31 (0.00–2.68)	3.99 (2.66–6.56)	< 0.001*	1.64 (1.17–3.77)	2,73 (0.00–5.01)	0.404
Final pathology results						
RP GGG			< 0.001*			0.409
1	20	2		14	8	
2	25	10		24	11	
3	0	9		8	1	
4	0	5		3	2	
5	0	4		4	0	
pT stage			< 0.001*			0.669
pT1- pT2	45	12		41	16	
pT3- pT4	0	18		12	6	
pN stage			0.003*			1.000
pN0-pNx	45	23		48	20	
pN1	0	7		5	2	

*AP* adverse pathology, *SD* standard deviation, *PSA* prostate-specific antigen, *IQR* interquartile range, *PV* prostate volume, *PSAD* prostate-specific antigen density, *FPSA* free prostate-specific antigen, *TPSA* total prostate-specific antigen, *mpMRI* GGG, Gleason Grade Group, *SUVmax* maximum standardized uptake values, *SUVmean* mean standardized uptake values, *PSMA-TL* prostate-specific membrane antigen total lesion, *PSMA-TV* prostate-specific membrane antigen tumour volume, *RP* radical prostatectomy

*P < 0.05

### Clinical model

The results of the clinical features in the comparison of the patients with AP and patients without AP are shown in Table 2. The univariate logistic regression analysis showed significant differences in FPSA/TPSA, SUVmax, SUVmean, PSMA-TL, and PSMA-TV between the two groups (*P* < 0.05). Subsequently, the significant variables from the univariate analysis were included in the multivariate logistic regression models. The results showed that FPSA/TPSA (odds ratio [OR]: 0.00, 95% confidence interval [CI]: 0.00–0.57) and PSMA-TV (OR: 1.29, 95% CI: 1.06–1.58) were the independent predictors for adverse pathology. Finally, the clinical model was established according to FPSA/TPSA and the PSMA-TV. As shown in Table 3, the AUC, sensitivity, and specificity of the training group were 0.821 (0.695–0.947), 76.2% (58.0%–94.4%), 81.2% (67.7%–94.8%), respectively, and 0.795 (0.603–0.987), 77.8% (50.6%-100%), 69.2% (42.4%-87.3%) in the testing group.

**Table 2:** Univariate and multivariate Logistic analysis of clinical factors for predicting patients with adverse pathology

	Univariate analysis		Multivariate analysis	
	OR (95% CI)	*P* value	OR (95% CI)	*P* value
Age (years)	0.96 (0.90–1.03)	0.270	-	-
PSA (ng/ml)	1.04 (0.99–1.09)	0.060	-	-
FPSA (ng/ml)	0.98 (0.85–1.14)	0.803	-	-
PV (ml)	1.01 (0.99–1.02)	0.337	-	-
PSAD (ng/ml^2^)	2.84 (0.88–9.16)	0.081	-	-
FPSA/TPSA	0.00 (0.00–0.27)	0.024*	0.00 (0.00–0.57)	0.037*
Biopsy GGG	1.79 (0.70–4.55)	0.220	-	-
% of positive cores	1.05 (0.36–3.08)	0.104	-	-
SUVmax	1.13 (1.03–1.24)	0.008*	1.70 (0.68–4.25)	0.064
SUVmean	1.22 (1.04–1.42)	0.015*	0.50 (0.10–2.47)	0.084
PSMA-TL	1.04 (1.01–1.08)	0.010*	0.98 (0.93–1.04)	0.444
PSMA-TV	1.32 (1.08–1.62)	0.007*	1.29 (1.06–1.58)	0.013*

*OR* odds ratio, *CI* confidence interval, *PSA* prostate-specific antigen, *FPSA* free prostate-specific antigen, *PV* prostate volume, *PSAD* prostate-specific antigen density, *TPSA* total prostate-specific antigen, *GGG* Gleason Grade Group, *SUVmax* maximum standardized uptake values, *SUVmean* mean standardized uptake values, *PSMA-TL* prostate-specific membrane antigen total lesion, *PSMA-TV* prostate-specific membrane antigen tumour volume

*P < 0.05

**Table 3:** Diagnostic performance of three models in training and testing cohorts

	Training cohort			Testing cohort		
	Clinical Model	Radiomics Model	Combined Model	Clinical Model	Radiomics Model	Combined Model
AUC	0.821 (0.695–0.947)	0.830 (0.720–0.941)	0.875 (0.780–0.970)	0.795 (0.603–0.987)	0.829 (0.624–1.000)	0.872 (0.678–1.000)
Sensitivity	76.2% (58.0%-94.4%)	90.5% (77.9%-100%)	90.5% (71.9%-100%)	77.8% (50.6%-100%)	77.8% (50.6%-100%)	88.9% (56.5%-99.4%)
Specificity	81.2% (67.7%-94.8%)	68.8% (52.7%-84.8%)	78.1% (61.3%-89.0%)	69.2% (42.4%-87.3%)	92.3% (77.8%-100%)	84.6% (57.8%-97.3%)
PPV	72.7% (54.1%-91.3%)	65.5% (48.2%-82.8%)	73.1% (53.9%-86.3%)	63.6% (35.4%-84.8%)	87.5% (64.6%-100%)	91.7% (68.6%-100%)
NPV	83.9% (70.9%-96.8%)	92.0% (75.0%-98.6%)	92.6% (76.6%-98.9%)	81.8% (62.3%-96.8%)	85.7% (67.4%-100%)	80.0% (61.0%-96.0%)

*AUC* area under curve, *PPV* positive predictive value, *NPV* negative predictive value

### Radiomics model

A total of 107 Image Biomarker Standardization Initiative (IBSI) compliant radiomic features were extracted from whole prostate PET images. Among them, 30 radiomic features were retained by mRMR. Then, the optimal adjustment weight λ (λ = 0.0672759851974577) was determined for the LASSO algorithm (Figure S1), and 6 nonzero coefficient features were selected to construct the final radiomic model. Figure S2 shows the detailed names and weights of the 6 radiomics features.

**Fig. 1:**
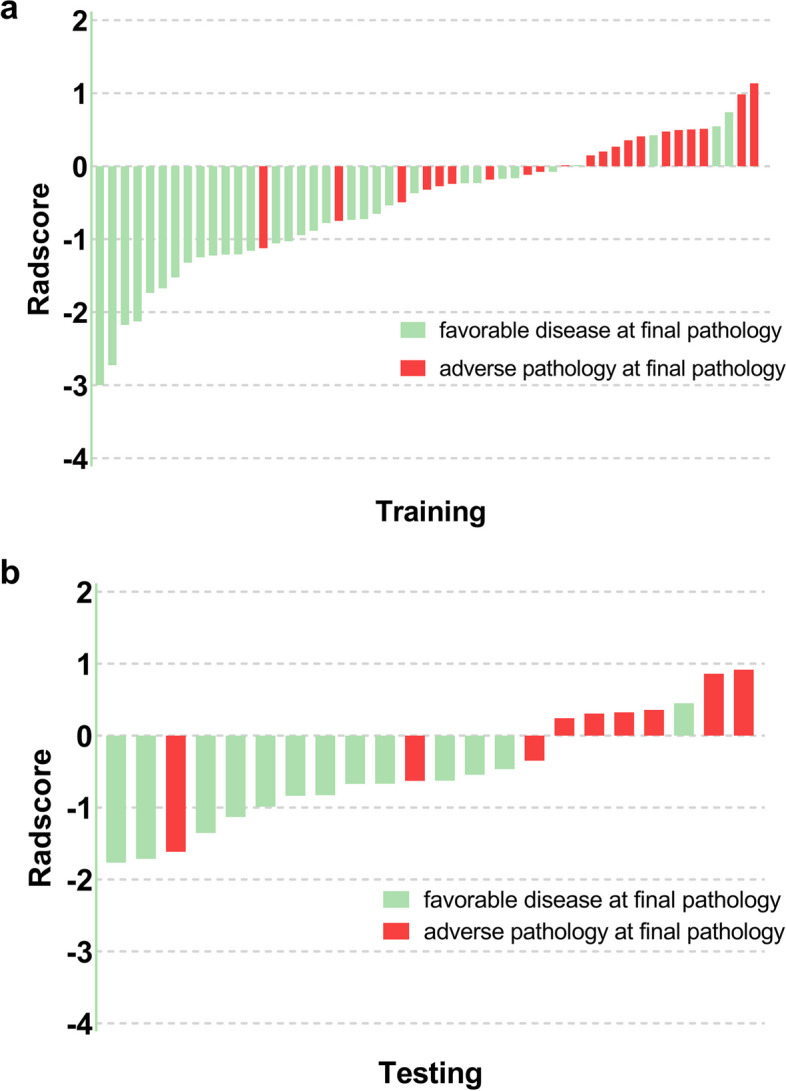
Bar diagrams of Radscore for each patient in the training cohort (**a**) and testing cohort (**b**). The red bars are Radscore values for patients with adverse pathology at final pathology, and the green bars are Radscore values for patients with favorable disease at final pathology. Radscore, radiomics score

Radscore was calculated by multiplying each feature coefficient by the corresponding eigenvalue and summing. The Radscores for all patients were shown in Fig. 1. In both training and testing cohorts, the patients with AP group had a higher Radscore than patients without, and the Radscores showed great discrimination performance to distinguish between these two groups. The radiomics model yielded an AUC, sensitivity, and specificity of 0.830 (0.720–0.941), 90.5% (77.9%-100%), and 68.8% (52.7%-84.8%) in the training group. In the testing group, the radiomics model demonstrated an equal sensitivity of 77.8% (50.6%-100%) and higher specificity (overlapping 95% CIs) of 92.3% (77.8%-100%) than a clinical model, with an AUC of 0.829 (0.624–1.000) (Table 3).

### Combined model

**Fig. 2:**
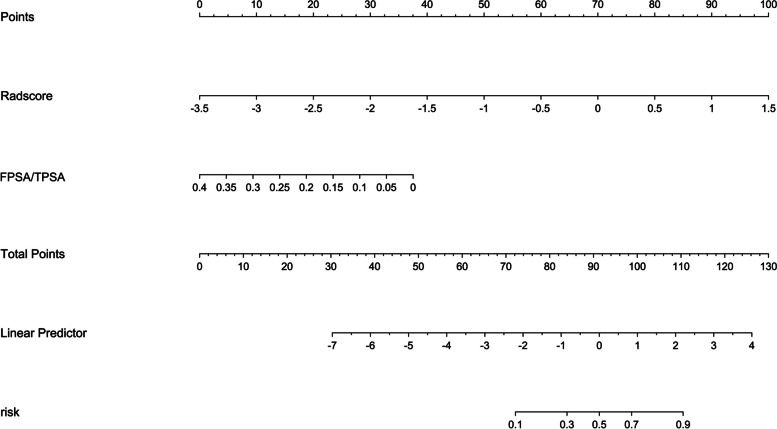
Nomogram based on the combined model predicting AP at RP, among patients with biopsy GGG 1–2 PCa. *AP* adverse pathology, *RP* radical prostatectomy, *GGG* Gleason Grade Group, *PCa* prostate cancer, *Radscore* radiomics score, *FPSA* free prostate-specific antigen, *TPSA* total prostate-specific antigen

Clinical features with statistically significant differences between the two groups and Radscore were included in multivariate logistic regression to establish a combined model. The results showed that FPSA/TPSA (OR: 0.00, 95% CI: 0.00–2.21) and Radscore (OR: 9.92, 95% CI: 2.72–36.23) were the significant independent predictors of AP. A nomogram including FPSA/TPSA, and Radscore based on the combined model was shown in Fig. 2. ROC curve analysis showed that the AUC values for the combined model were 0.875 (0.780–0.970) and 0.872 (0.678–1.000) in the training and test cohorts, respectively, showing good sensitivity and specificity (Table 3).

### The comparison and evaluation of the three models

**Fig. 3:**
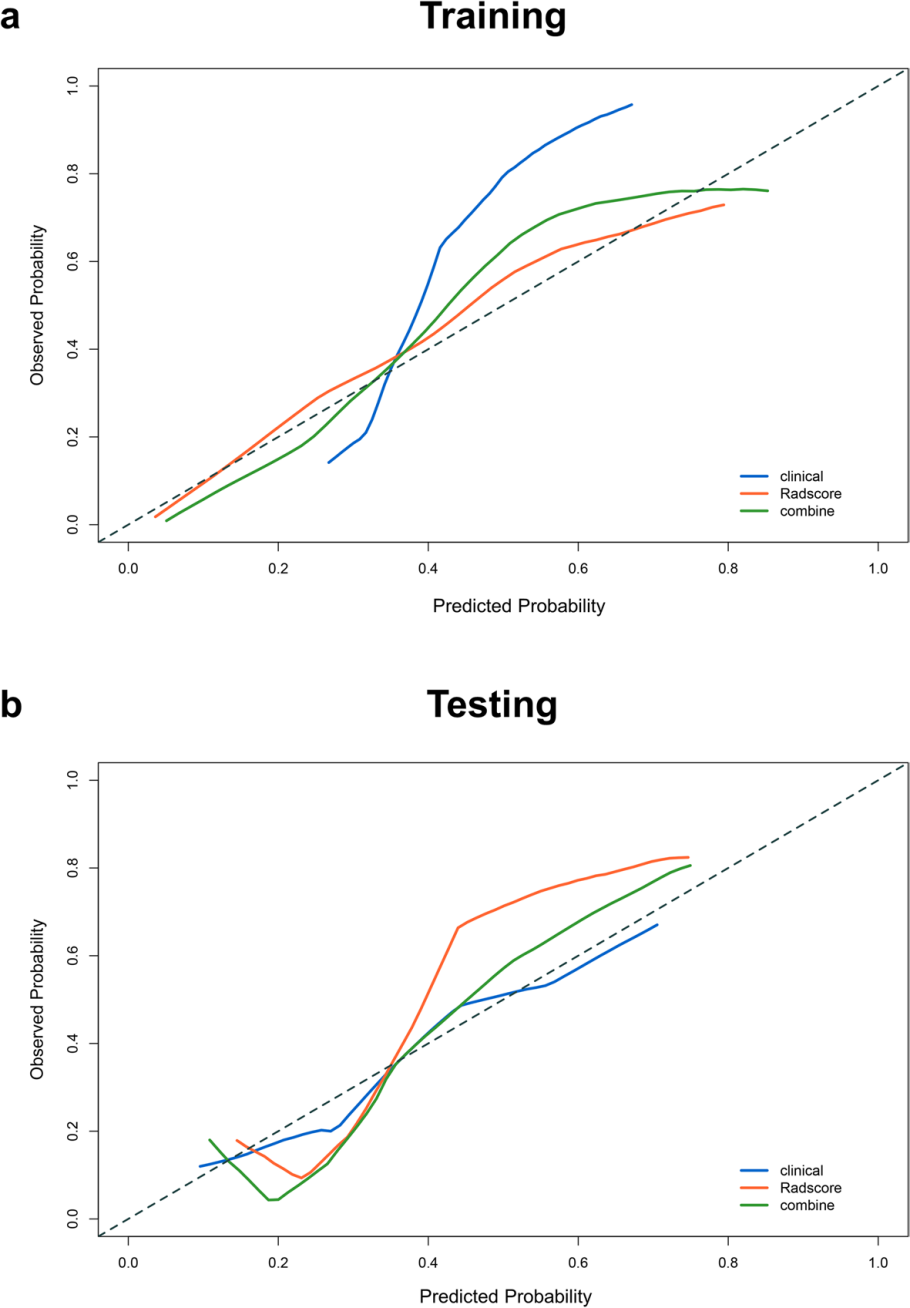
The calibration curves of the three models in the training cohort (**a**) and testing cohort (**b**). The *P*-values of the clinical model, radiomics model, combined model were 0.303, 0.593, 0.445 in the training group, and 0.465, 0.598, 0.685 in the testing group

**Fig. 4:**
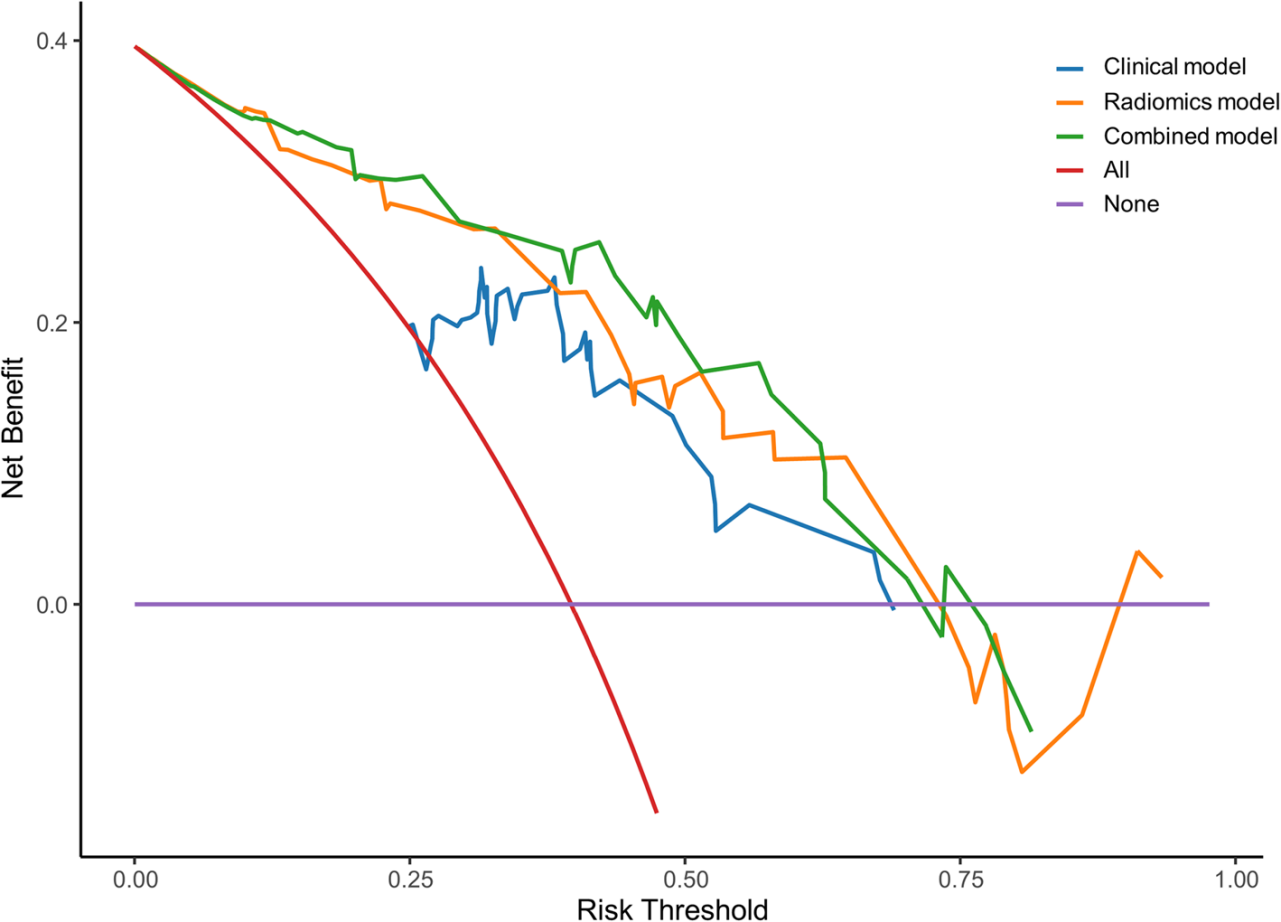
DCA of the clinical, radiomics, and combined models for predicting AP at final pathology in the testing cohort. *DCA* decision curve analysis, *AP* adverse pathology

A comparison of the ROC curves of these three models is shown in Figure S3. The combined model displayed the highest AUC (overlapping 95% CIs) values among the three models, with the highest sensitivity (overlapping 95% CIs) and moderate specificity in both training and testing cohorts. The Hosmer–Lemeshow calibration curves for the three predictive models were constructed in the training and testing groups. If the predicted probabilities on the calibration curve closely resembled the observed probabilities, and the *P*-value of the Hosmer–Lemeshow test was greater than 0.05, it indicated a high calibration accuracy of the model. In our study, it clearly demonstrated a high degree of concordance between the dotted lines (reference lines) and the coloured lines (calibration curve) in Fig. 3. In addition, the *P*-values of the clinical model, radiomics model, combined model were 0.303, 0.593, 0.445 in the training group, and 0.465, 0.598, 0.685 in the testing group. These results showed good agreement between the predicted and actual results. As shown in Fig. 4, DCA was performed to compare the clinical utility of the three prediction models in predicting the AP. The results indicated that the net benefit of the combined model and the radiomics model was greater than that of the clinical model.

## Discussion

In this study, we developed three models based on clinical and/or [^68^Ga]Ga-PSMA PET-based radiomics features to redefine the inclusion criteria for AS in patients with biopsy Gleason Grade Group 1–2 PCa, which may have important clinical value in maximally reducing overtreatment and avoiding inappropriate adverse pathology patients progressing. The combined model demonstrated superior predictive ability compared to the clinical and radiomics models alone, specifically in identifying patients with adverse pathology at the final analysis, who should not be considered for AS. Internal validation results revealed that the combined model effectively fulfills the clinical requirements for selecting appropriate AS candidates, leveraging the full potential of [^68^Ga]Ga-PSMA PET/CT scans.

Currently, guidelines stratify PCa patients based on biopsy results, PSA levels, and clinical stage, with AS primarily recommended for low-risk patients [8]. However, the strict inclusion criteria of AS limit the inclusion of suitable patients. Moreover, the limited number of included indicators omits some patient information, leading to the inclusion of patients who may not be appropriate for AS, resulting in delays in their treatment [16, 31]. To extend the AS inclusion criteria and reduce the inclusion of unsuitable patients, several predictive models based on clinical characteristics and conventional imaging features have been developed in recent years. Gandaglia et al. [32] developed a multivariable model using patients' PSA levels, clinical stage, biopsy grade group, number of positive cores, and PSA density to assess the risk of poor outcomes in low-risk or intermediate-risk PCa patients, aiding in the selection of AS candidates. The results demonstrated a 10% increase in the number of patients eligible for AS compared to PRIAS criteria, without increasing the risk of misclassification [33]. However, the diagnostic efficacy of this model remains poor, possibly due to the absence of specific imaging characteristics from prostate MRI and [^68^Ga]Ga-PSMA PET/CT. Another study, which developed a multivariable model including variables from MRI and targeted biopsy, validated that the inclusion of MRI features significantly enhanced the diagnostic performance of the model for adverse pathology [16]. This improvement may be attributed to the correlation between PI-RADS scores and adverse pathology, as well as the more accurate pathology obtained through MRI-targeted biopsy. Previous studies have shown that [^68^Ga]Ga-PSMA PET/CT is a more accurate predictor of adverse pathological outcomes compared to mpMRI [15, 34]. In addition, previous literatures have proved that PSMA PET-targeted biopsy, combined with the technique of intraoperative quantification of PSMA PET uptake in core biopsies, could improve the detection rate of csPCa compared with systematic biopsy and reduce the need for saturation biopsy [35]. In our study, we developed a clinical model based on patients' clinical characteristics and conventional PSMA PET/CT features (FPSA/TPSA and PSMA-TV), which demonstrated improved diagnostic performance. However, it is undeniable that the lesion features provided by visual assessment of PSMA PET/CT are limited, and the acquisition of some features is subjective.

Radiomics can extract features in a high-throughput and quantitative manner that cannot be obtained through visual evaluation by clinicians. This can improve the accuracy of diagnosis, prognosis, and prediction [36, 37]. Currently, research on utilizing radiomics data to select patients for AS is limited and predominantly based on MRI imaging [38–40]. Compared to models using clinical and imaging visual evaluation features, radiomics models based on MRI imaging often exhibit similar or slightly lower diagnostic performance [38, 39]. This discrepancy may stem from the inherent challenge of MRI images in differentiating various PCa pathologies. Considering the potential of PET-derived radiomics as biomarkers for predicting treatment outcomes and characterizing tumor biology in a non-invasive manner is noteworthy [41]. Specifically, radiomic features derived from [^68^Ga]Ga-PSMA-11 PET/CT images have shown remarkable proficiency in discerning Gleason scores [24]. Our study presents the pioneering application of [^68^Ga]Ga-PSMA PET radiomics in selecting patients for AS. Encouragingly, our results demonstrate that the radiomics model based on PSMA PET imaging outperforms clinical models in terms of diagnostic performance in both the training and testing sets, confirming the ability of [^68^Ga]Ga-PSMA PET to identify adverse postoperative pathology in patients with AS.

Advances in technology have revolutionized the management of PCa, with mounting evidence supporting the adoption of sophisticated tests and comprehensive features to individualize patient assessment and ensure optimal treatment [42]. The proposed radiomics-based analysis incorporating the clinical-radiographic feature could provide a noninvasive biomarker for the individualized and precise medical treatment of patients [40]. In our study, we developed a predictive model incorporating PSMA PET imaging, histology features, and FPSA/TPSA, which exhibited superior diagnostic performance compared to both clinical and imaging histology models, with consistent results in the test set. This underscores the complementary nature of clinical and imaging histology features and the increased robustness achieved through their combination. Current major clinical guidelines, including the European Association of Urology (EAU) guideline, recommend active surveillance as the treatment of choice for patients with low-risk prostate cancer. In our internal validation queue, the use of our combined model to select AS candidates would allow for an 83.3% increase in the number of patients eligible for AS without increasing the risk of adverse pathological characteristics compared to the EAU criteria. Our combined model also showed excellent calibration characteristics at internal validation. Notably, among patients with a predicted risk of AP greater than 40%, the model would underestimate the actual risk of AP. Some individuals with AP might receive an AS regimen. Among patients with a predicted risk of AP less than 40%, the model would overestimate the actual risk of AP. Some individuals without AP might excluded from an AS regimen. Furthermore, we employed nomogram plots to enhance the visualization and clinical utility of the model, providing a clear representation of the impact of each factor on the target event for individual patients. In clinical practice, these nomogram plots can facilitate the scoring of patients based on their clinical and imaging histology features, enabling the assessment of their probability of harboring adverse pathology. As a result, they provide guidance to clinicians in selecting appropriate AS patients. Radiomics features are arguably more dependent on the underlying image data. A recent meta-analysis suggests that [^68^Ga]Ga-PSMA and [^18^F]F-DCFPyL PET have comparable diagnostic performance in patients with suspected prostate cancer [43]. A study including 160 men found that the SUVmax of [^18^F]F-PSMA and [^68^Ga]Ga-PSMA did not differ (*P* > 0.05) in local recurrence or primary prostate cancer [44]. These results seem to indicate that there is no difference in the uptake of [^18^F]- and [^68^Ga]-labeled PSMA ligands in prostate cancer PET scans. However, the [^68^Ga]-labeled PSMA ligand used in these studies is [^68^Ga]Ga PSMA-11, and there is limited data on [^68^Ga]Ga-PSMA-617. In addition, there are no studies that have investigated whether the value of PET-based radiomics features is valid for different PSMA ligands. Further multicentre, large-scale studies will be required to establish with certainty the accuracy and wider applicability of the radiomic signature proposed here.

Several limitations are evident in this study. Firstly, the sample size was relatively small due to strict inclusion criteria. And external validation is lacking in the present study, which may restrict the generalizability of our results. Future validation would benefit from additional multicenter, large-scale studies. Secondly, the study's endpoint of AP at RP is a surrogate outcome for cancer-specific survival in AS patients. However, this limitation is common in most studies as intermediate-risk patients are usually offered active treatment. Finally, this study did not incorporate MRI-related visual assessments and imaging histology features because the MRI examinations for most patients were conducted at external hospitals, which could result in substantial differences between MRI image acquisition and interpretation.

## Conclusions

In conclusion, we have developed the first model based on the PSMA PET-derived radiomics and clinical features in identifying candidates for AS, which has the potential to aid in the safe selection of Gleason Grade Group 1–2 patients for AS to increase the absolute proportion of men eligible for AS and decrease their risk of overtreatment.

### Supplementary Information

Supplementary Material 1.

## Data Availability

No datasets were generated or analysed during the current study.

## Abbreviations

[^68^Ga]Ga-PSMA: [^68^Ga]-labeled prostate-specific membrane antigen
AP: Adverse pathology
AS: Active surveillance
AUC: Area under the curve
CI: Confidence interval
DCA: Decision curve analysis
FBW: Fixed bin width
FPSA: Free prostate-specific antigen
GGG: Gleason Grade Group
GLCM: Gray Level Co-occurrence Matrix
GLDM: Gray Level Dependence Matrix
GLRLM: Gray Level Run Length Matrix
GLSZM: Gray Level Size Zone Matrix
IBSI: Image Biomarker Standardization Initiative
ISUP: International Society of Urologic Pathology
LASSO: Least absolute shrinkage and selection operator
mpMRI: Multiparametric magnetic resonance imaging
mRMR: Minimum redundancy maximum relevance
NGTDM: Neighbouring Gray Tone Difference Matrix
OR: Odds ratio
PCa: Prostate cancer
PET/CT: Positron emission tomography/computed tomography
pN: Pathological N stage
PSA: Prostate-specific antigen
PSAD: Prostate-specific antigen density
PSMA-TL: Prostate-specific membrane antigen total lesion
PSMA-TV: Prostate-specific membrane antigen tumor volume
pT: Pathological T stage
Radscore: Radiomics score
ROC: Receiver operating characteristic
RP: Radical prostatectomy
SUVmax: Maximal standardized uptake value
SUVmean: Mean standardized uptake value
TPSA: Total prostate-specific antigen density
TRUS: Transrectal ultrasound
VOI: Volume of interest

## References

[1] Moschini M, Carroll PR, Eggener SE, Epstein JI, Graefen M, Montironi R (2017). Low-risk prostate cancer: identification, management, and outcomes. Eur Urol.

[2] Briganti A, Fossati N, Catto JWF, Cornford P, Montorsi F, Mottet N (2018). Active surveillance for low-risk prostate cancer: the European Association of Urology position in 2018. Eur Urol.

[3] Carlsson S, Benfante N, Alvim R, Sjoberg DD, Vickers A, Reuter VE (2020). Long-term outcomes of active surveillance for prostate cancer: the Memorial Sloan Kettering Cancer Center experience. J Urol.

[4] Philippou Y, Raja H, Gnanapragasam VJ (2015). Active surveillance of prostate cancer: a questionnaire survey of urologists, clinical oncologists and urology nurse specialists across three cancer networks in the United Kingdom. BMC Urol.

[5] Bruinsma SM, Bangma CH, Carroll PR, Leapman MS, Rannikko A, Petrides N (2016). Active surveillance for prostate cancer: a narrative review of clinical guidelines. Nat Rev Urol.

[6] Barrett T, Haider MA (2017). The emerging role of MRI in prostate cancer active surveillance and ongoing challenges. AJR Am J Roentgenol.

[7] Van Hemelrijck M, Ji X, Helleman J, Roobol MJ, van der Linden W, Nieboer D (2019). Reasons for discontinuing active surveillance: assessment of 21 centres in 12 countries in the Movember GAP3 Consortium. Eur Urol.

[8] Lam TBL, MacLennan S, Willemse PM, Mason MD, Plass K, Shepherd R (2019). EAU-EANM-ESTRO-ESUR-SIOG prostate cancer guideline panel consensus statements for deferred treatment with curative intent for localised prostate cancer from an international collaborative study (DETECTIVE Study). Eur Urol.

[9] Bokhorst LP, Valdagni R, Rannikko A, Kakehi Y, Pickles T, Bangma CH (2016). A decade of active surveillance in the PRIAS study: an update and evaluation of the criteria used to recommend a switch to active treatment. Eur Urol.

[10] Klotz L, Vesprini D, Sethukavalan P, Jethava V, Zhang L, Jain S (2015). Long-term follow-up of a large active surveillance cohort of patients with prostate cancer. J Clin Oncol.

[11] Filippou P, Welty CJ, Cowan JE, Perez N, Shinohara K, Carroll PR (2015). Immediate versus delayed radical prostatectomy: updated outcomes following active surveillance of prostate cancer. Eur Urol.

[12] Conti SL, Dall'era M, Fradet V, Cowan JE, Simko J, Carroll PR (2009). Pathological outcomes of candidates for active surveillance of prostate cancer. J Urol.

[13] Morlacco A, Cheville JC, Rangel LJ, Gearman DJ, Karnes RJ (2017). Adverse disease features in Gleason score 3 + 4 "favorable intermediate-risk" prostate cancer: implications for active surveillance. Eur Urol.

[14] Suardi N, Briganti A, Gallina A, Salonia A, Karakiewicz PI, Capitanio U (2010). Testing the most stringent criteria for selection of candidates for active surveillance in patients with low-risk prostate cancer. BJU Int.

[15] Akcay K, Kibar A, Sahin OE, Demirbilek M, Beydagi G, Asa S (2024). Prediction of clinically significant prostate cancer by [(68) Ga]Ga-PSMA-11 PET/CT: a potential tool for selecting patients for active surveillance. Eur J Nucl Med Mol Imaging.

[16] Lantz A, Falagario UG, Ratnani P, Jambor I, Dovey Z, Martini A (2022). Expanding active surveillance inclusion criteria: a novel nomogram including preoperative clinical parameters and magnetic resonance imaging findings. Eur Urol Oncol.

[17] Panebianco V, Giganti F, Kitzing YX, Cornud F, Campa R, De Rubeis G (2018). An update of pitfalls in prostate mpMRI: a practical approach through the lens of PI-RADS v. 2 guidelines. Insights Imaging.

[18] Sonn GA, Fan RE, Ghanouni P, Wang NN, Brooks JD, Loening AM (2019). Prostate magnetic resonance imaging interpretation varies substantially across radiologists. Eur Urol Focus.

[19] Liu J, Wang ZQ, Li M, Zhou MY, Yu YF, Zhan WW (2020). Establishment of two new predictive models for prostate cancer to determine whether to require prostate biopsy when the PSA level is in the diagnostic gray zone (4–10 ng ml(-1)). Asian J Androl.

[20] Fendler WP, Eiber M, Beheshti M, Bomanji J, Calais J, Ceci F (2023). PSMA PET/CT: joint EANM procedure guideline/SNMMI procedure standard for prostate cancer imaging 2.0. Eur J Nucl Med Mol Imaging..

[21] Hofman MS, Lawrentschuk N, Francis RJ, Tang C, Vela I, Thomas P (2020). Prostate-specific membrane antigen PET-CT in patients with high-risk prostate cancer before curative-intent surgery or radiotherapy (proPSMA): a prospective, randomised, multicentre study. Lancet.

[22] Hupe MC, Philippi C, Roth D, Kümpers C, Ribbat-Idel J, Becker F (2018). Expression of prostate-specific membrane antigen (PSMA) on biopsies is an independent risk stratifier of prostate cancer patients at time of initial diagnosis. Front Oncol.

[23] Zamboglou C, Bettermann AS, Gratzke C, Mix M, Ruf J, Kiefer S (2021). Uncovering the invisible-prevalence, characteristics, and radiomics feature-based detection of visually undetectable intraprostatic tumor lesions in (68)GaPSMA-11 PET images of patients with primary prostate cancer. Eur J Nucl Med Mol Imaging.

[24] Zamboglou C, Carles M, Fechter T, Kiefer S, Reichel K, Fassbender TF (2019). Radiomic features from PSMA PET for non-invasive intraprostatic tumor discrimination and characterization in patients with intermediate- and high-risk prostate cancer - a comparison study with histology reference. Theranostics.

[25] Ghezzo S, Mapelli P, Bezzi C, Samanes Gajate AM, Brembilla G, Gotuzzo I (2023). Role of [(68)Ga]Ga-PSMA-11 PET radiomics to predict post-surgical ISUP grade in primary prostate cancer. Eur J Nucl Med Mol Imaging.

[26] Draulans C, De Roover R, van der Heide UA, Kerkmeijer L, Smeenk RJ, Pos F (2021). Optimal (68)Ga-PSMA and (18)F-PSMA PET window levelling for gross tumour volume delineation in primary prostate cancer. Eur J Nucl Med Mol Imaging.

[27] Iczkowski KA, van Leenders G, van der Kwast TH (2021). The 2019 International Society of Urological Pathology (ISUP) consensus conference on grading of prostatic carcinoma. Am J Surg Pathol.

[28] Amin MB, Greene FL, Edge SB, Compton CC, Gershenwald JE, Brookland RK (2017). The Eighth Edition AJCC cancer staging manual: continuing to build a bridge from a population-based to a more "personalized" approach to cancer staging. CA Cancer J Clin.

[29] Zwanenburg A, Vallières M, Abdalah MA, Aerts H, Andrearczyk V, Apte A (2020). The image biomarker standardization initiative: standardized quantitative radiomics for high-throughput image-based phenotyping. Radiology.

[30] Ding C, Peng H (2005). Minimum redundancy feature selection from microarray gene expression data. J Bioinform Comput Biol.

[31] Iremashvili V, Manoharan M, Parekh DJ, Punnen S (2016). Can nomograms improve our ability to select candidates for active surveillance for prostate cancer?. Prostate Cancer Prostatic Dis.

[32] Gandaglia G, van den Bergh RCN, Tilki D, Fossati N, Ost P, Surcel CI (2018). How can we expand active surveillance criteria in patients with low- and intermediate-risk prostate cancer without increasing the risk of misclassification? Development of a novel risk calculator. BJU Int.

[33] Bul M, Zhu X, Valdagni R, Pickles T, Kakehi Y, Rannikko A (2013). Active surveillance for low-risk prostate cancer worldwide: the PRIAS study. Eur Urol.

[34] Roberts MJ, Morton A, Donato P, Kyle S, Pattison DA, Thomas P (2021). (68)Ga-PSMA PET/CT tumour intensity pre-operatively predicts adverse pathological outcomes and progression-free survival in localised prostate cancer. Eur J Nucl Med Mol Imaging.

[35] Ferraro DA, Laudicella R, Zeimpekis K, Mebert I, Müller J, Maurer A (2022). Hot needles can confirm accurate lesion sampling intraoperatively using [(18)F]PSMA-1007 PET/CT-guided biopsy in patients with suspected prostate cancer. Eur J Nucl Med Mol Imaging.

[36] Lv L, Xin B, Hao Y, Yang Z, Xu J, Wang L (2022). Radiomic analysis for predicting prognosis of colorectal cancer from preoperative (18)F-FDG PET/CT. J Transl Med.

[37] Wu G, Woodruff HC, Shen J, Refaee T, Sanduleanu S, Ibrahim A (2020). Diagnosis of invasive lung adenocarcinoma based on chest CT radiomic features of part-solid pulmonary nodules: a multicenter study. Radiology.

[38] Sushentsev N, Rundo L, Blyuss O, Gnanapragasam VJ, Sala E, Barrett T (2021). MRI-derived radiomics model for baseline prediction of prostate cancer progression on active surveillance. Sci Rep.

[39] Sushentsev N, Rundo L, Blyuss O, Nazarenko T, Suvorov A, Gnanapragasam VJ (2022). Comparative performance of MRI-derived PRECISE scores and delta-radiomics models for the prediction of prostate cancer progression in patients on active surveillance. Eur Radiol.

[40] Sushentsev N, Rundo L, Abrego L, Li Z, Nazarenko T, Warren AY (2023). Time series radiomics for the prediction of prostate cancer progression in patients on active surveillance. Eur Radiol.

[41] Cook GJR, Azad G, Owczarczyk K, Siddique M, Goh V (2018). Challenges and promises of PET radiomics. Int J Radiat Oncol Biol Phys.

[42] Williams IS, McVey A, Perera S, O'Brien JS, Kostos L, Chen K (2022). Modern paradigms for prostate cancer detection and management. Med J Aust.

[43] Jiang Z, Guo J, Hu L, Yang S, Meng B, Tang Q (2024). Diagnostic performance of (18)F-DCFPyL PET vs. (68)Ga-PSMA PET/CT in patients with suspected prostate cancer: a systemic review and meta-analysis. Oncol Lett.

[44] Kroenke M, Mirzoyan L, Horn T, Peeken JC, Wurzer A, Wester HJ (2021). Matched-pair comparison of (68)Ga-PSMA-11 and (18)F-rhPSMA-7 PET/CT in patients with primary and biochemical recurrence of prostate cancer: frequency of non-tumor-related uptake and tumor positivity. J Nucl Med.

