# Supplementary material for: [68Ga]Ga‑PSMA‑617 PET-based radiomics model to identify candidates for active surveillance amongst patients with GGG 1–2 prostate cancer at biopsy
Source: Cancer Imaging. 2024 Jul 4;24:86. doi: 10.1186/s40644-024-00735-2 (PMC11229016; doi:10.1186/s40644-024-00735-2)
Supplement: Supplementary file 1 — Supplementary Material 1. [file 40644_2024_735_MOESM1_ESM.docx]

**Supplementary Information for:**

**[^68^Ga]Ga‑PSMA‑617 PET-based radiomics model to identify candidates for active surveillance amongst patients with Gleason Grade Group 1-2 prostate cancer at biopsy**

**Authors:** Jinhui Yang MD^1†^, Ling Xiao MD^1†^, Ming Zhou PharM^1^, Yujia Li MD^1^, Yi Cai PhD^2,3^, Yu Gan PhD^2,3*^, Yongxiang Tang PhD^1,3,4*^, Shuo Hu PhD^1,3,5*^

**Affiliations:**

^1^ Department of Nuclear Medicine, Xiangya Hospital, Central South University, Changsha, Hunan, China.

^2^ Department of Urology, Disorders of Prostate Cancer Multidisciplinary Team, Xiangya Hospital, Central South University, Changsha, Hunan, China

^3^ National Clinical Research Center for Geriatric Disorders (XIANGYA), Xiangya Hospital, Central South University, Changsha, Hunan, China.

^4^ Department of Nuclear Medicine, Inselspital, University Hospital Bern, Bern, Switzerland

^5^ Key Laboratory of Biological Nanotechnology of National Health Commission, Xiangya Hospital, Central South University, Changsha, Hunan, China.

**† Co-first authors:** Jinhui Yang and Ling Xiao contributed equally to this work.

**Corresponding author:**

Shuo Hu, Department of Nuclear Medicine, XiangYa Hospital, Central South University, 87 Xiangya Road, Changsha, Hunan 410008, China. Tele: +86-731-89753869; Fax: +86-0731-89753869; E-mail: [hushuo2018@163.com](mailto:hushuo2018@163.com)

Yongxiang Tang, Department of Nuclear Medicine, XiangYa Hospital, Central South University, 87 Xiangya Road, Changsha, Hunan 410008, China. E-mail: 405035@csu.edu.cn

Yu Gan, Department of Urology, Disorders of Prostate Cancer Multidisciplinary Team, National Clinical Research Center for Geriatric Disorders, Xiangya Hospital, Central South University, 87 Xiangya Road, Changsha, Hunan 410008, China. E-mail: 148302039@csu.edu.cn


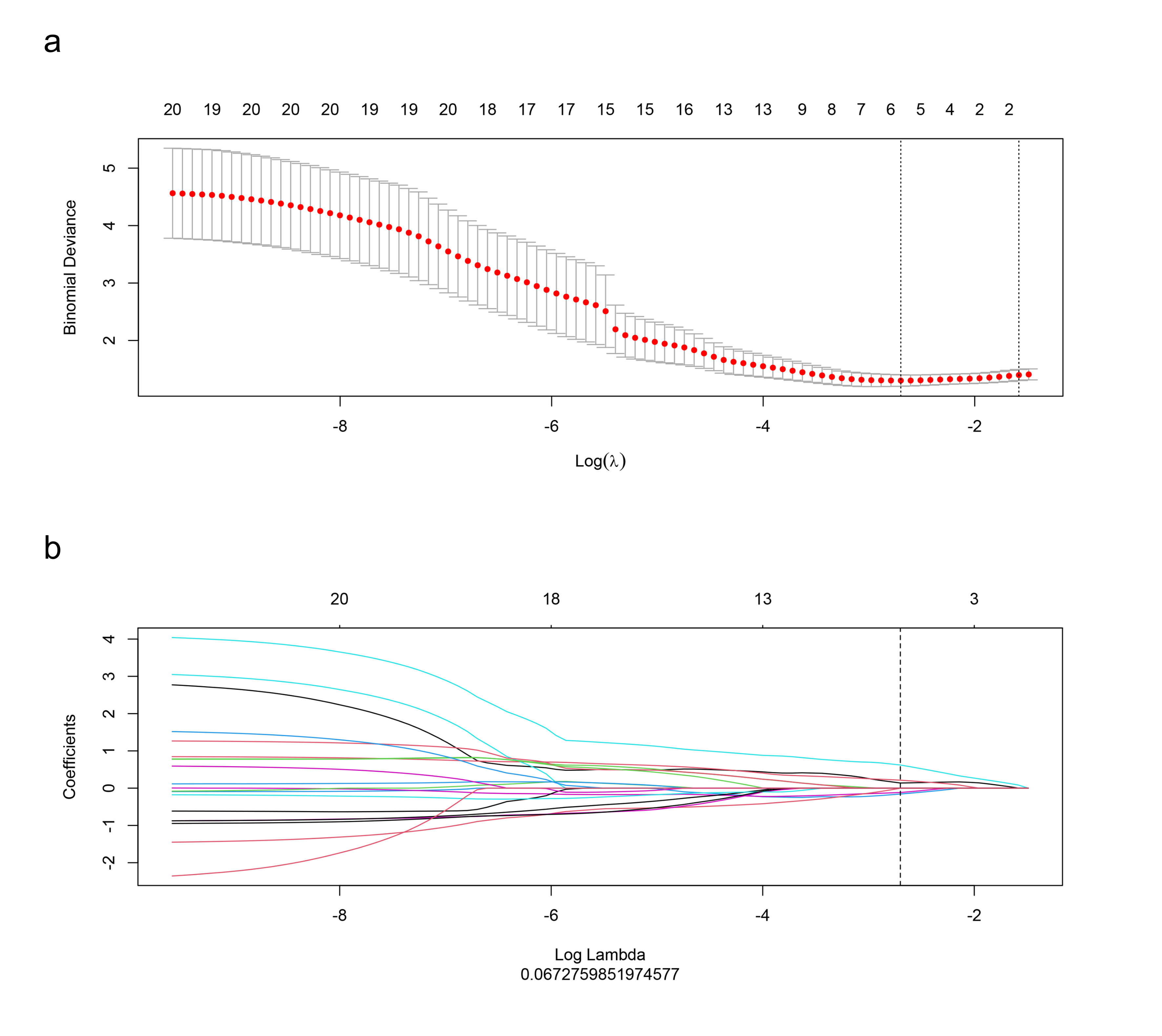


**Figure S1.** Radiomics feature selection using the least absolute shrinkage and selection operator (LASSO) algorithm. (a) Selection of the regulation weight λ. (b) The LASSO coefficient profiles of the features.


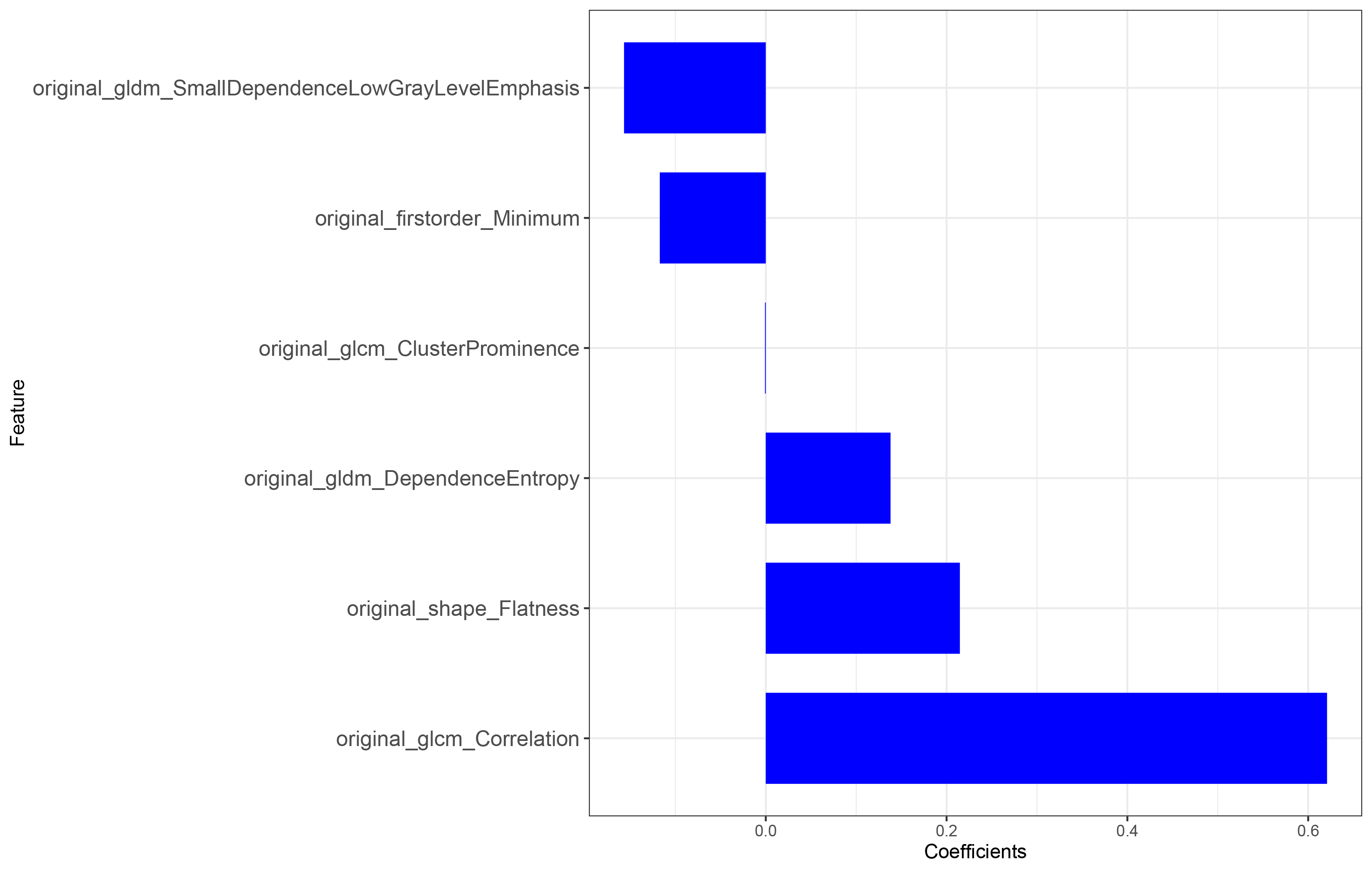


**Figure S2.** The weights of radiomics features selected.

**
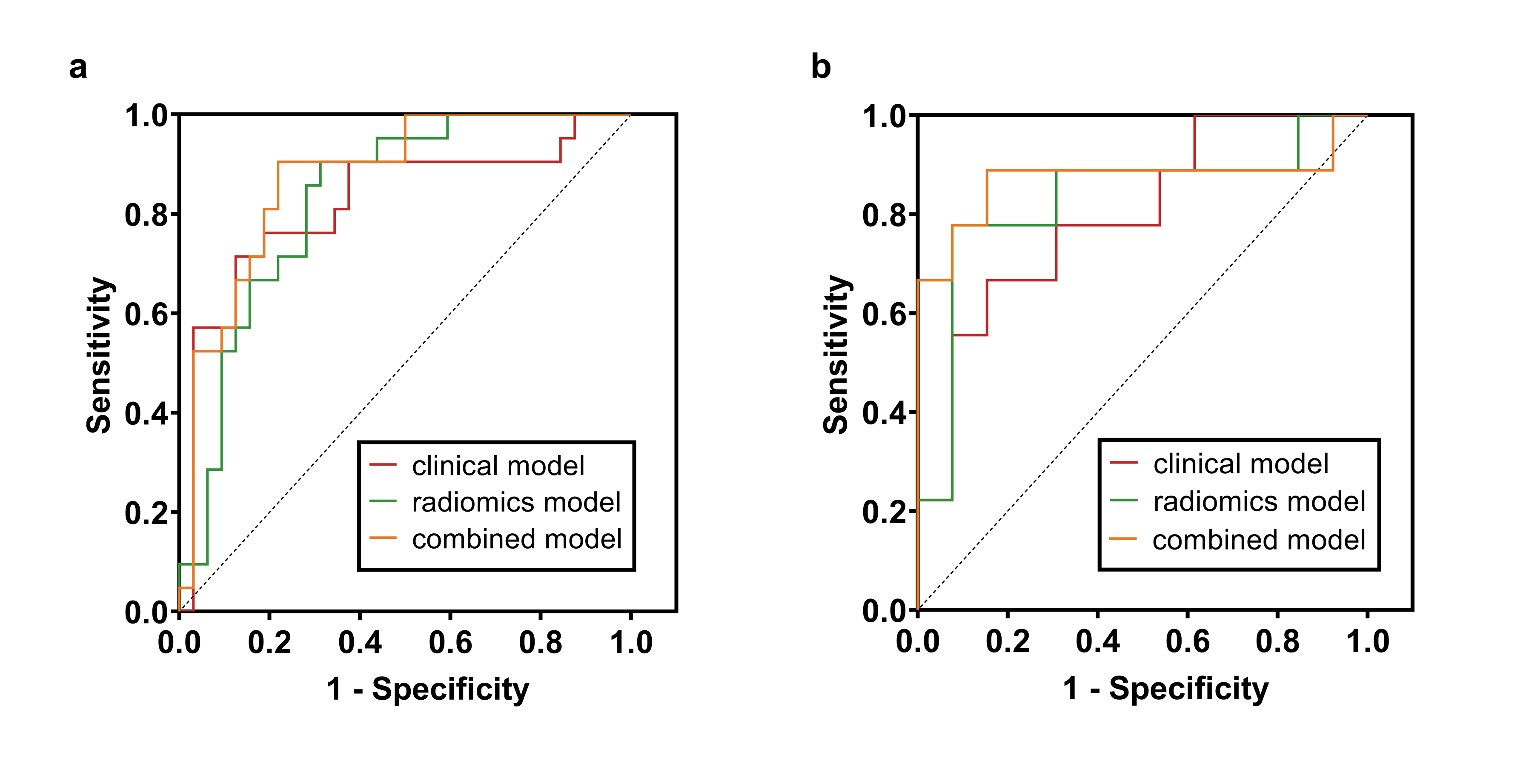
Figure S3** ROC of the three models for predicting AP at final pathology in the training cohort (a) and testing cohort (b). ROC, Receiver operating characteristic; AP, adverse pathology.
